# Complete genome sequences of two effective nitrogen-fixing *Sinorhizobium medicae* strains isolated from *Medicago sativa* and *Melilotus albus* in Canada

**DOI:** 10.1128/mra.00105-26

**Published:** 2026-04-01

**Authors:** Eden S. P. Bromfield, Sylvie Cloutier

**Affiliations:** 1 Agriculture and Agri-Food Canada, Ottawa, Canada; Indiana University Bloomington, Bloomington, Indiana, USA

**Keywords:** *Sinorhizobium medicae* strains T2 and T10, complete genome sequences, Canada, *Medicago sativa*, *Melilotus albus*

## Abstract

We report complete genome sequences of nitrogen-fixing *Sinorhizobium medicae* strains T2 and T10 isolated from *Medicago* and *Melilotus*. Each genome comprises a chromosome (~4.1 to 5.5 Mb) and three plasmids (~0.17 to 1.58 Mb) encoding predicted nodulation and nitrogen-fixation genes but not predicted type III secretion system genes.

## ANNOUNCEMENT

*Sinorhizobium medicae* is a bacterial species that elicits nitrogen-fixing symbioses predominantly with annual *Medicago* species native to the Mediterranean basin (1). Strains T2 and T10 were isolated in 1992 from root nodules of *Medicago sativa* (alfalfa) and *Melilotus albus* (white sweet clover) grown at a Canadian field site (45.3851° N, 75.7043° W) (2, 3). Nodules were surface-sterilized (70% ethanol, 2.5% sodium hypochlorite), rinsed, and crushed (2). Suspensions were streaked onto tryptone-yeast-extract (TY) agar (4). Pure cultures obtained from single colonies were maintained in 20% glycerol at −80°C. Both strains were identified as *S. medicae* by analysis of housekeeping gene sequences and showed high nitrogen-fixation effectiveness in plant assays with *M. sativa* and *M. albus* (3).

For genomic analysis, bacteria grown on TY agar for 3 days at 28°C were harvested by gentle scraping, washed with nuclease-free water, and pelleted by centrifugation. DNA was extracted using the Wizard SV system (Promega) and purified using DNeasy PowerClean Pro Kit (Qiagen). Strain T2 DNA was sheared (Covaris g-TUBE) without size selection for continuous long read (CLR) sequencing; strain T10 was prepared without shearing for circular consensus sequencing (CCS).

Libraries were constructed using the SMRTbell Express Template Prep Kit 2.0 (Pacific Biosciences, California, USA) and sequenced on a PacBio Sequel II platform at Genome Québec, Canada. Raw data were processed using SMRT Link v10.1 (5) for adapter trimming and quality filtering of CLR subreads (strain T2) and generating error-corrected circular consensus (HiFi) reads (strain T10). *De novo* assembly was performed using Flye (v2.9.0) (6). Circularity was confirmed by identifying and trimming overlapping contig ends. Assembly quality was assessed with CheckM (v1.2.3) (7) using the *Sinorhizobium medicae* marker set, and genomes were annotated with NCBI PGAP (v6.10) (8). Sequencing metrics are summarized in Table 1.

**TABLE 1:** Characteristics of the complete genome sequences of *Sinorhizobium medicae* strains T2 and T10 compared with reference strain WSM1115^*a*^

Characteristic		T2	T10	WSM1115
Sequencing metrics				
Total raw (polymerase) reads, assembly coverage		3,816,296; 3,049×	1,702,673; 10,645×	na
Filtered subreads (number; mean [bp]; N50 [bp])		3,816,296; 7,052; 9,821	17,026,728; 5,060; 7,235	na
Genome characteristics				
Genome size (bp) (no. of contigs)		7,417,501 (4)	7,073,256 (4)	7,063,185 (4)
Replicon size (bp) (accession no.)	Chromosome	4,138,833 (CP149869)	5,514,198 (CP149881)	4,106,266 (CP088109)
	Plasmid a	462,704 (CP149872)	173,406 (CP149880)	276,847 (CP088112)
	Plasmid b	1,232,518 (CP149870)	189,288 (CP149882)	1,128,391 (CP088111)
	Plasmid c	1,583,446 (CP149871)	1,196,364 (CP149879)	1,551,681 (CP088110)
G + C content (%)		61.1	61.1	61.2
Annotation features				
Total predicted genes		7,255	6,868	6,814
Total predicted protein-coding genes (CDSs)		6,760	6,451	6,453
Predicted rRNAs (5S, 23S, 16S)		3 , 3, 3 (in chromosome)	3 , 3, 3 (in chromosome)	3 , 3, 3 (in chromosome)
Predicted tRNAs		57	55	56
Predicted nodulation genes		*nodD1D2D3ABCQIEFGHJP*, *noeAB*, *nolFGN*	*nodD1D2D3ABCQIEFGHJPL*, *noeAB*, *nolFGN*	*nodD1D2D3ABCQIEFGHJPL*, *noeAB*, *nolFGN*
Predicted nitrogen-fixation genes		*nifHDKENBUXAT*, *fixABCX*, *fixNOQP*, *fixGHIS*, *fixKLJU*, *fdxNB*, *rpoN*, *modABC*, *sufS*	*ifHDKENBUXAT*, *fixABCX*, *fixNOQP*, *fixGHIS*, *fixKLJU*, *fdxNB*, *rpoN*, *modABC*, *sufS*	*nifHDKENBUXAT*, *fixABCX*, *fixNOQP*, *fixGHIS*, *fixKLJU*, *fdxNB*, *rpoN*, *modABC*, *sufS*
Predicted type IV secretion system genes		*TraG*/*VirD4*, *TrbBCDEFGIJL*, *VirB1-* *5*, *7–11*	*TraG*/*VirD4*, *TrbBCDEFGIJL*, *VirB1-* *5*, *7–11*	*TraG*/*VirD4*, *TrbBCDEFGIJL*, *VirB1-5*, *7–11*
Predicted Insertion sequences		204	141	101

^*a*^ Symbiosis plasmids are indicated by accession numbers shown in bold. Raw reads are total PacBio Sequel II polymerase reads; coverage (×) is based on total raw bases divided by genome size. Filtered subreads are adapter and quality trimmed reads used for assembly; mean and N50 were calculated from filtered subreads. na, not applicable.

**Fig 1:**
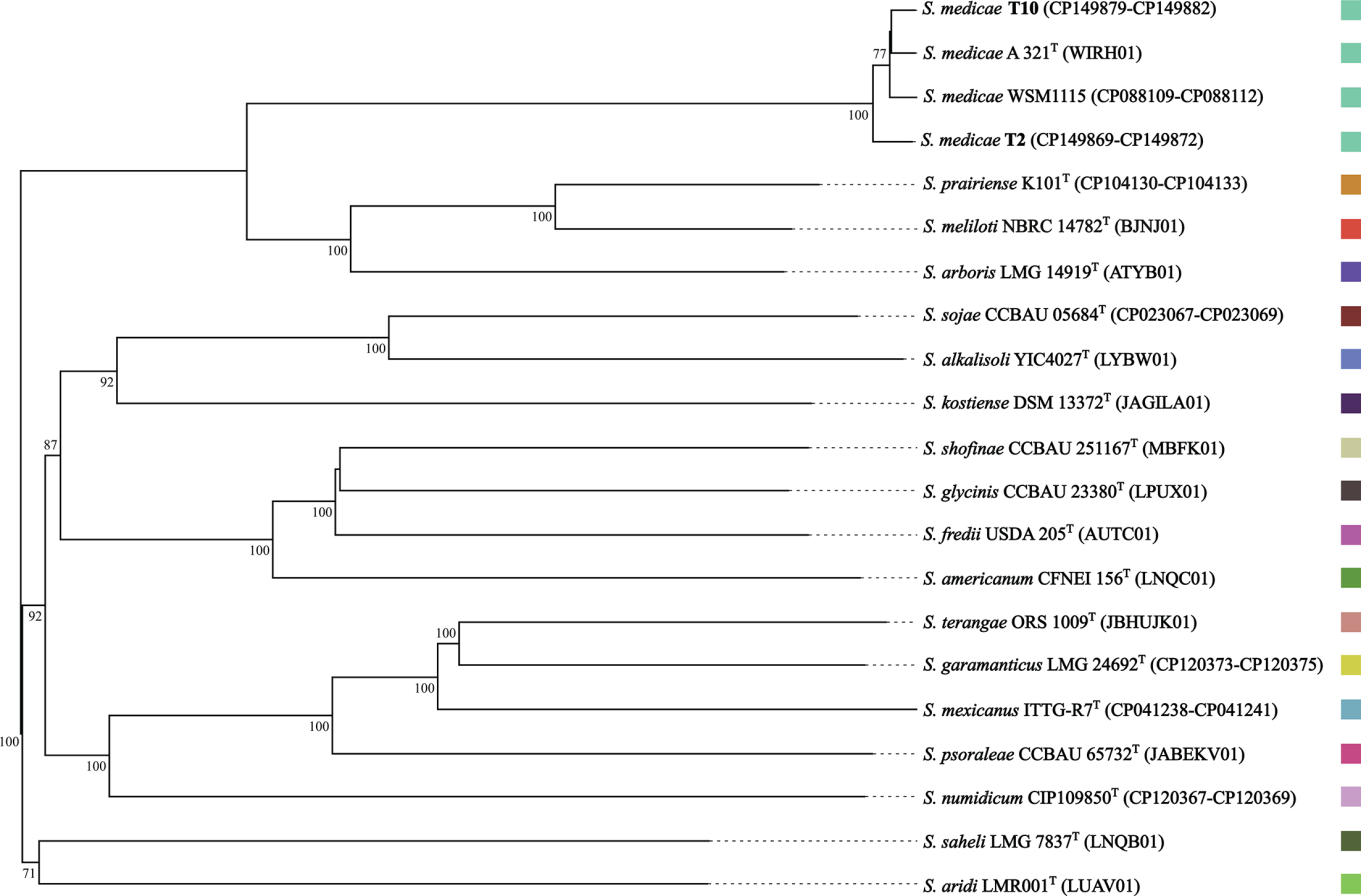
Phylogenomic tree inferred using FastME (v2.1.6.1) from Genome BLAST Distance Phylogeny (GBDP) distances generated through the TYGS pipeline (10), showing the placement of *Sinorhizobium medicae* strains T2 and T10 relative to reference taxa of the genus *Sinorhizobium*. Branch lengths are scaled according to the GBDP distance formula d5. Numbers above branches represent GBDP pseudo-bootstrap support values based on 100 replications. Solid colors denote TYGS species assignments; strains sharing the same color are assigned to the same species. Sequence accession numbers are given in brackets.

FastANI v1.34 (9) values for strains T2 and T10 versus the species type strain A321^T^ (1) were 99.02% and 99.23%, respectively, confirming their assignment to *S. medicae*. Species affiliation was corroborated by placement of T2 and T10 in a whole genome-based TYGS (v403) tree inferred using FastME (v2.1.6.1) from Genome BLAST Distance Phylogeny distances (10) (Fig. 1).

Table 1 shows the genomic features of T2 and T10 compared with reference strain *S. medicae* WSM1115. The genome of each novel strain comprises a chromosome (~4.1 to 5.5 Mb) and three plasmids (~0.17 to 1.58 Mb); plasmids were identified by the presence of predicted *repABC* genes (11). Each strain harbors a symbiosis megaplasmid containing predicted core genes for nodulation and nitrogen fixation. However, predicted type III secretion system genes, required for nodulation by some other rhizobia (12), were absent.

Predicted genes for conjugative type IV secretion systems (13) implicated in horizontal gene transfer and competitiveness for nodule occupancy were detected in all strains.

Numerous insertion sequences predicted with ISEScan (v1.7.3) (14) were present in all strains, suggesting genome plasticity and environmental adaptability (15).

These high-quality assemblies serve as a valuable resource for further genomic studies with effective nitrogen-fixing *S. medicae* showing potential for sustainable agriculture.

## Data Availability

The whole genome shotgun projects for *S. medicae* strains T2 and T10 were deposited at DDBJ/ENA/GenBank as assembly accession numbers GCA_017571595.2 and GCA_040114925.1, respectively. Raw PacBio data were deposited in the NCBI Sequence Read Archive as SRX25642144 and SRX25717176 in BioProject accession numbers PRJNA715162 and PRJNA715171, respectively. *S. medicae* strains T2 and T10 were deposited in the BCCM/LMG Bacteria Collection, University of Ghent, Belgium, under the culture collection numbers LMG 32372 and LMG 32373, respectively.
